# Nanozymes: A New Disease Imaging Strategy

**DOI:** 10.3389/fbioe.2020.00015

**Published:** 2020-02-06

**Authors:** Peixia Wang, Tao Wang, Juanji Hong, Xiyun Yan, Minmin Liang

**Affiliations:** ^1^CAS Engineering Laboratory for Nanozyme, Key Laboratory of Protein and Peptide Pharmaceutical, Institute of Biophysics, Chinese Academy of Sciences, Beijing, China; ^2^Experimental Center of Advanced Materials School of Materials Science & Engineering, School of Materials Science & Engineering, Beijing Institute of Technology, Beijing, China; ^3^College of Life Sciences, University of Chinese Academy of Sciences, Beijing, China; ^4^Department of Neurosurgery, Peking University Third Hospital, Beijing, China

**Keywords:** nanozyme, natural enzyme, disease imaging, precision medicine, tumor

## Abstract

Nanozymes are nanomaterials with intrinsic enzyme-like properties. They can specifically catalyze substrates of natural enzymes under physiological condition with similar catalytic mechanism and kinetics. Compared to natural enzymes, nanozymes exhibit the unique advantages including high catalytic activity, low cost, high stability, easy mass production, and tunable activity. In addition, as a new type of artificial enzymes, nanozymes not only have the enzyme-like catalytic activity, but also exhibit the unique physicochemical properties of nanomaterials, such as photothermal properties, superparamagnetism, and fluorescence, etc. By combining the unique physicochemical properties and enzyme-like catalytic activities, nanozymes have been widely developed for *in vitro* detection and *in vivo* disease monitoring and treatment. Here we mainly summarized the applications of nanozymes for disease imaging and detection to explore their potential application in disease diagnosis and precision medicine.

## Graphical Abstract

Nanozymes for disease imaging and diagnosis. Adapted with permission from Fan et al. (2012), Li et al. (2017), Ding et al. (2019).

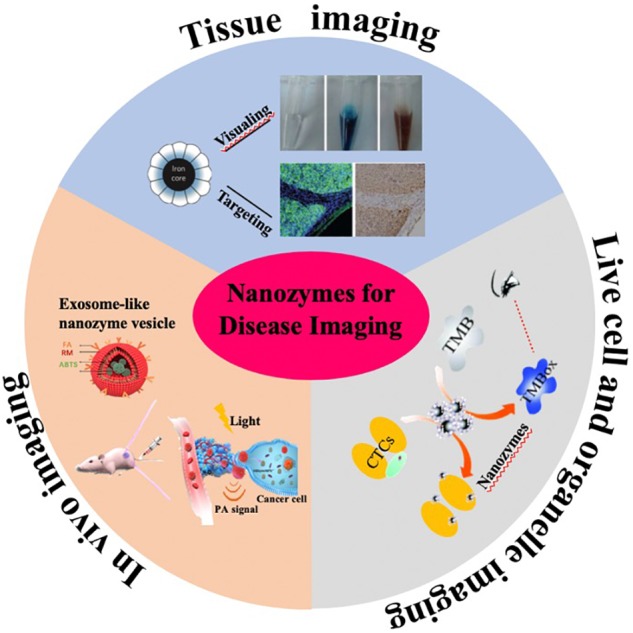

## Introduction

Nanozyme is a new type of artificial enzyme with intrinsic enzyme-like characteristics. In 2007, we reported the landmark paper that Fe_3_O_4_ nanoparticles (NPs) have intrinsic peroxidase-like activity (Gao et al., 2007), and since that time nanozymes have increasingly attracted attention from a broad spectrum of scientists and technologists because of their high catalytic activity, low cost, and high stability (Gao and Yan, 2016). To date, there are more than 300 types of nanomaterials that have been found to possess the intrinsic enzyme-like activity, including the peroxidase activity of Fe_3_O_4_ (Gao et al., 2007), Co_3_O_4_ (Mu et al., 2012), CuO (Liu et al., 2014), V_2_O_5_ (André et al., 2011), MnFeO_3_ (Chi et al., 2018), FeS (Dai et al., 2009), graphene quantum dots (Nirala et al., 2017), CeO_2_ (Xue et al., 2012), BiFeO_3_ (Wei et al., 2010), CoFe_2_O_4_ (He et al., 2010), FeTe (Roy et al., 2012), *g*old@carbon dots (Zheng et al., 2016); oxidase activity of Au (Comotti et al., 2004), Pt (Yu et al., 2014), CoFe_2_O_4_ (Zhang et al., 2013), MnO_2_ (Xing et al., 2012), CuO NPs (Hu et al., 2017) and NiCo_2_O_4_ (Su et al., 2017); catalase activity of CeO_2_ NPs (Talib et al., 2010), Pt-Ft NPs (Fan et al., 2011), Ir NPs (Su et al., 2015), MoS_2_ nanosheets (Chen et al., 2018), Prussian Blue NPs (Zhang W. et al., 2016); superoxide oxidase activity of CeO_2_ (Tarnuzzer et al., 2005), Fullerene (Ali et al., 2004), FePO_4_ microflowers (Wang W. et al., 2012), Gly-Cu (OH)_2_ NPs (Korschelt et al., 2017), N-PCNs (Fan et al., 2018); haloperoxidase activity of V_2_O_5_ nanowire (Natalio et al., 2012), CeO_2__x_ Nanorods (Herget et al., 2017); sulfite oxidase activity of MoO_3_ NPs (Ragg et al., 2014); phosphatase activity of CeO_2_ (Kuchma et al., 2010), Fe_2_O_3_ NPs (Huang, 2018); phosphotriesterase activity of Co_3_O_4_/GO nanocomposites (Wang et al., 2017), CeO_2_ NPs (Vernekar et al., 2016), MOF-808 (M = Zr) (Mondal and Holdt, 2016), UiO-66@LiOtBu (M = Zr) (Mondal and Holdt, 2016); CO oxidase activity of Cu_2_O@CeO_2_ core@shell nanocubes (Wang et al., 2015); chymotrypsin activity of Cr-MIL-101 (Nath et al., 2016); G-selective DNA cleaving activity of fullerene carboxylic acid (Tokuyama et al., 1993); protease activity of Cu-MOF (Li et al., 2014); restriction endonuclease activity of CdTe NPs (Sun et al., 2018); carbonic anhydrase activity of Co-BBP@Tb-MOF (Sahoo et al., 2013), etc. With the emergence of the new concept of “nanozymology” (Jiang B. et al., 2018), “Nanozymes” have now become an emerging new field bridging nanotechnology and biology.

As a new type of promising artificial enzymes, nanozymes have shown a broad spectrum of applications because of their obvious advantages including high stability, high catalytic activity, low cost, large surface area for functionalization, and tunable activity. In particular, by combining their unique physicochemical properties (such as fluorescence, X-ray absorption, and paramagnetic properties, etc.), nanozymes have been widely explored from *in vitro* detection (Leng et al., 2009; Liang et al., 2010; Roy et al., 2012) to *in vivo* disease imaging and therapy (Hyon Bin et al., 2010; Yang et al., 2012; Kwon et al., 2016; Singh et al., 2017; Fan et al., 2018). In this review, we summarized the progress of nanozymes in disease detection and imaging, and discussed the current challenges and future directions of nanozyme development in disease imaging and diagnosis.

## Nanozymes for Pathological Disease Diagnosis

**FIGURE 1:**
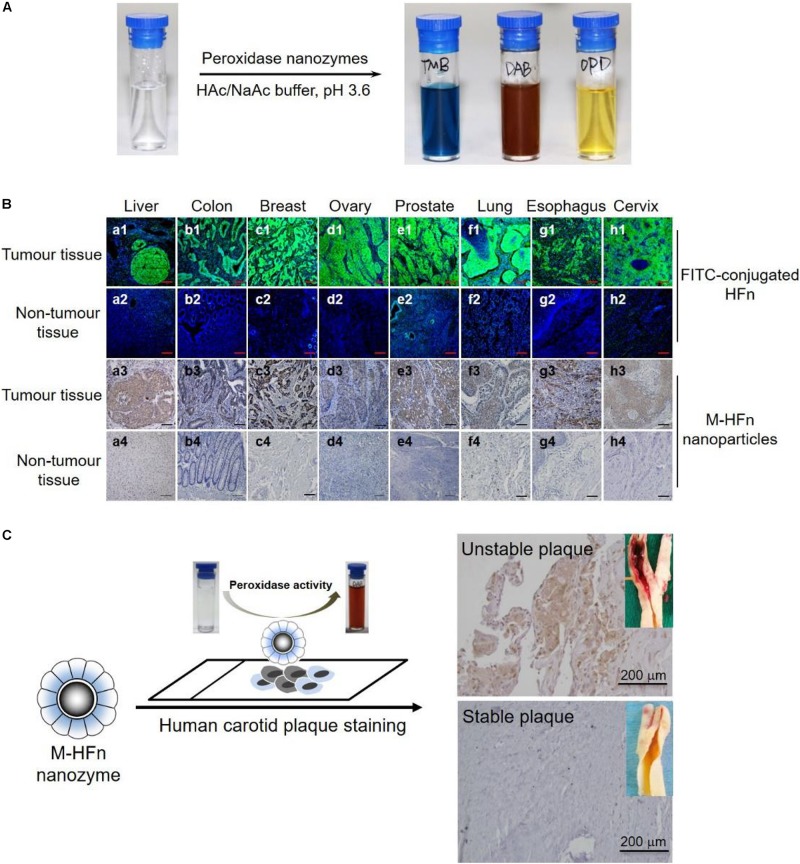
Nanozymes for pathological tissue imaging. **(A)** Peroxidase nanozymes catalyze the oxidation of various peroxidase substrates (TMB, DAB, and OPD) in the presence of H_2_O_2_ to produce different color reactions. Adapted with permission from ref (Jiang B. et al., 2018), © 2018, Springer Nature. **(B)** M-HFn nanozymes specifically stained tumor tissues from different organs. Adapted with permission from ref (Fan et al., 2012), © 2012, Springer Nature. **(C)** Peroxidase nanozymes for the pathological identification of unstable atherosclerotic plaques from patients with symptomatic carotid disease. Reproduced with permission from ref (Liang and Yan, 2019), © 2019, American Chemical Society.

Peroxidase nanozymes catalyze the oxidation of colorimetric substrates, such as 3,3,5,5-tetramethylbenzidine (TMB), diazo-aminobenzene (DAB), and o-phenylenediamine (OPD), to give a color reaction that can be used for imaging the recognized biomarkers within tissue sections for pathological disease diagnosis (Figure 1A). In 2012, Our group developed a magnetoferritin nanozyme (M-HFn) which is composed of a recombinant human heavy-chain ferritin (HFn) protein nanocage encapsulated an iron oxide nanocore for tumor targeting and imaging (Fan et al., 2012). HFn nanocage specifically recognized tumor cells via binding to overexpressed transferrin receptor1 (TfR1) in tumor cells. Iron oxide nanocores catalyzed the oxidation of color substrates in the presence of H_2_O_2_ to produce an intense color reaction for visualizing tumor tissues. We examined 474 clinical human specimens including 247 clinical tumor tissues and 227 normal tissues and demonstrated that M-HFn nanozymes could identify nine types of cancer cells with a specificity of over 95% and sensitivity of 98%. The concentration of M-HFn was 1.8 μM, and the reactive time was 1 h for DAB staining (Figure 1B). Likewise, Gu’s groups developed avastin antibody-functionalized Co_3_O_4_ nanozymes as target-specific peroxidase mimics for immunohistochemical staining of vascular endothelial growth factor (VEGF) in tumor tissues and the concentration of Ab-Co_3_O_4_ was 15 μg/ml, 100 μL, and the reactive time was 30 min for DAB staining (Dong et al., 2014). Due to the high peroxidase-like activity, Co_3_O_4_ nanozyme has been proved to be a potential label in place of natural enzymes. So far, numerous of peroxidase nanozyme-based staining methods have been developed for pathological diagnosis of breast cancer, colorectal, stomach, and pancreas (Zhang T. et al., 2016), hepatocellular carcinoma (Hu et al., 2014; Jiang et al., 2019), esophageal cancer (Wu et al., 2011), and bladder cancer (Peng et al., 2019).

By compare with the traditional immunohistochemistry, the nanozyme-based pathological staining method is more rapid and sensitive because of their higher catalytic activity than natural enzymes [e.g., horseradish peroxidase (HRP)], which greatly shortens the diagnostic time and reduces the cost and thus has significant implications for clinical pathological diagnosis. In addition, besides tumor pathological diagnosis, peroxidase nanozymes have also been used for pathological identification of human high-risk and ruptured atherosclerotic plaques (Wang et al., 2019). M-HFn nanozymes specifically distinguish the ruptured and high-risk plaque tissues via TfR1, which is highly expressed in plaque-infiltrated macrophages and significantly associated with the increasing risk of plaque rupture. As shown Figure 1C, M-HFn peroxidase nanozymes could specifically distinguish high-risk plaque tissues from patients with symptomatic carotid disease, and M-HFn staining showed a significant correlation with plaque vulnerability (*r* = 0.89, *P* < 0.0001).

**FIGURE 2:**
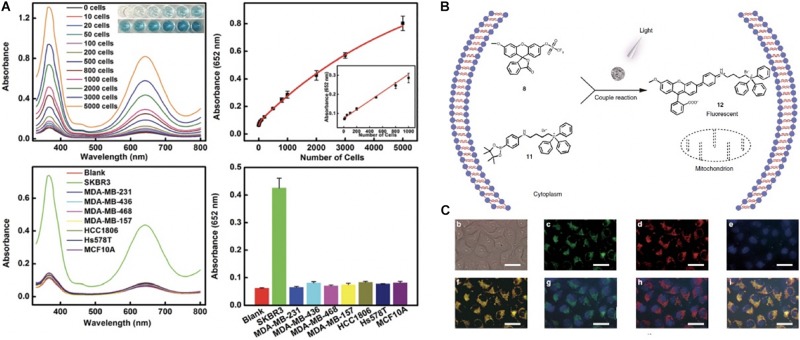
Nanozymes for live cell and organelle imaging. **(A)** A multifunctional mesoporous silica-gold nanozyme platform for selective breast cancer cell detection using a catalytic amplification-based colorimetric assay. Reproduced with permission from ref (Li et al., 2019), © 2019, The Royal Society of Chemistry. **(B)** Scheme of nanozyme-based methods for mitochondrial fluorescent imaging. **(C)** Representative nanozyme-based light-mediated reversible catalysis for mitochondrial imaging. Adapted with permission from ref (Wang et al., 2018).

To further improve the detection sensitivity of nanozyme-based pathological staining method, much effort has been expended to improve the enzyme-like catalytic activity of nanozymes, including adjusting their size, shape, composition, surface modification, and heteroatomic doping (Dong et al., 2014; Zhang et al., 2017; Jiang et al., 2019; Li et al., 2019). In 2018, Leong and co-workers engineered a mesoporous silica-gold nanocluster hybrid nanozymes with excellent peroxidase-like catalytic activity for selective detection of HER2-positive (HER^2+^) breast cancer cell (Li et al., 2019). Owing to their high catalytic performance, the prepared silica-gold hybrid nanozymes achieved the detection limit of 10 cells using colorimetric analysis. The hybrid nanozymes did not stain, or only slightly stained, normal or lesion tissues, but strongly stained cancerous regions. Significantly, there was a clear distinction between cancerous cells and adjacent normal cells in representative sections (Figure 2A).

Besides enzyme-like activity, the unique physicochemical properties (such as luminescence, X-ray absorption, and paramagnetic properties) of nanozymes also have been widely developed for pathological tissue imaging. For instance, Cai’s group developed a folate receptor-targeting gold nanocluster as fluorescence enzyme mimetic nanoprobes for tumor tissues fluorescence visualizing detection. In the work, the intravenous dose used was 500 mg/kg for fluorescence imaging, and the concentration of 1.8 mM, 1 h was used for DAB staining (Hu et al., 2014). For the same tumor tissue slice, nanozyme staining and fluorescent staining were obtained simultaneously in a one-step incubation, and the results were mutually complementary. Thus, the developed fluorescence/nanozyme nanoprobes could provide a molecular colocalization diagnosis strategy within clinical tissue specimens, which efficiently avoids false-positive and false-negative results, and greatly improves the detection accuracy, credibility, and repeatability for cancer pathological diagnoses. Likewise, Zhang et al., also developed a gold nanozyme-based dark-field imaging assay as a novel immunohistochemical method for detecting HER2 overexpressed in breast cancer tissues (Lin et al., 2016). By quantitative analysis of the optical property of dark-field imaging, cancerous tissue can be quantitatively divided into four levels: “−, +, ++, and ++.”

Despite the fact that nanozyme-based staining methods have been broadly developed for pathological disease diagnosis, there are still many unresolved issues and challenges. The first is how to improve the enzyme-like activity of nanozymes. Since the catalytic activity of nanozymes is directly correlated with their detection sensitivity, the improvement of enzyme-like activity of nanozymes could help substantially improve the detection sensitivity of nanozyme-based staining methods. However, the issue of false positives would arise along with the improved enzyme-like activity (Wu et al., 2019). In addition, the false positive issue would become even more severe due to the limited substrate specificity of nanozymes. We proposed a strategy to improve both the catalytic activity and the substrate specificity by introducing histidine residues onto the surface of Fe_3_O_4_ nanozymes to mimic the natural peroxidase enzymes (Fan et al., 2017). Juewen Liu engineered a specific nanozyme using molecular imprinting method to enhance the substrate selectivity and activity of enzyme mimics (Zhang et al., 2017). In addition, the oriented immobilization of the recognizing moieties to the surface of nanozymes could also reduce false positives greatly. Guo et al., constructed an inorganic/protein hybrid nanozyme by oriented immobilizing nature enzymes on the surface of inorganic graphene NPs (Liu Y. et al., 2019). The prepared nanohybrid nanozymes exhibited outstanding peroxidase-mimicking activity and excellent substrate selectivity. The second challenge for nanozyme staining method is how to quantically analyze the pathological staining results. Currently, the clinical pathological analysis mainly relies on experienced judgment, which is subjective, and variation between observers is high for certain categories of pathological diagnosis (Qian and Jiao, 2017). By combining the unique optical property and enzyme-like catalytic activities, nanozymes hold promise to achieve quantitative analysis for pathological disease staining diagnosis.

## Nanozymes for Live Cell and Organelle Imaging

Cytological examination is an important means of clinical disease diagnosis (Bromberg et al., 2007; Mosterd et al., 2008; Hao et al., 2011; Venugopal and Prasad, 2015). Exfoliated cells from blood, cerebrospinal fluid, spinal fluid, chest water and mucous liquid can provide a large amount of clinical information (including cell morphology, cell type, and cell proportion, etc.), which can be used for cancer screening (Schiffman et al., 2000; Dillner et al., 2008), CNS hematologic malignancies (Bromberg et al., 2007) anemia diagnosis (Hao et al., 2011), and Langerhans cell granulomatosis detection (Mosterd et al., 2008).

Currently, the most commonly used cytological detection methods are flow cytometry, cytological smear and nucleic acid testing. These traditional methods are characterized by high technical requirements, time consuming or high cost. Nanozymes-driven color reaction can be used for qualitative and quantitative analysis of cytological features. Trau et al. extended the application of nanozymes to the detection of circulating tumor cells (CTCs) (Li et al., 2017). The targeting antibody-conjugated Fe_3_O_4_ nanozymes simultaneously achieved CTC magnetic isolation and visualization by catalyzing the oxidation of colorimetric substrate TMB into blue colored products. In addition, the visualized CTCs can be further quantified using UV-vis measurement. The developed nanozyme platform successfully detected 13 melanoma CTCs per mL blood within 50 min, and the concentration of Fe_3_O_4_ nanozymes used was about 0.2 mg/ml for TMB colorimetric development. Later, Wang et al. (2018) also developed an Fe_3_O_4_ NPs-based ultrasensitive electrochemical CTCs detection strategy (Tian et al., 2018). Under the optimized experimental conditions, the proposed nanozyme cytosensor exhibited significant analytical performance for MCF-7 CTCs detection with a detection limit of 6 cells mL^–1^ with a linear range from 15 to 45 cells mL^–1^ at the acceptable stability condition and reproducibility. Recently, nanozyme-based detection strategies have been broadly developed for the cytological detection of breast cancer cell (Li et al., 2019), cervical cancer cells (Yu et al., 2013; Maji et al., 2015), human chronic myelogenous leukemia cell (Ge et al., 2014), melanoma tumor cell (Li et al., 2017), and squamous cancer (Wang et al., 2014) etc.

In addition to detecting CTCs, researchers also employed the catalytic activity of nanozymes to design real-time detection probes for organelle imaging in living cells. For example, Qu et al., designed a heterogeneous palladium nanozyme that could effectively mediate the bioorthogonal reactions *in situ* through light and thus realized the specific imaging of mitochondria in living cells (Wang et al., 2018) (Figures 2B,C). Beside CTCs and organelle imaging detection, there are also several other nanozymes-based colorimetric methods for specific disease imaging, including jaundice (Santhosh et al., 2014), acquired immune deficiency syndrome (Lin et al., 2017), diabetes (Tianran et al., 2014), infectious disease (Kim et al., 2014; Duan et al., 2015), and neurodegenerative disease (Wang C. I. et al., 2012; Farhadi et al., 2014). Thus, compared to traditional methods (such as PCR, cell flow cytometry, and ELISA), nanozymes methods exhibit more broaden prospect for live cell and organelle imaging because nanozyme assay is more fast, cost-effective and much easier to operate.

## Nanozymes for *In Vivo* Imaging

**FIGURE 3:**
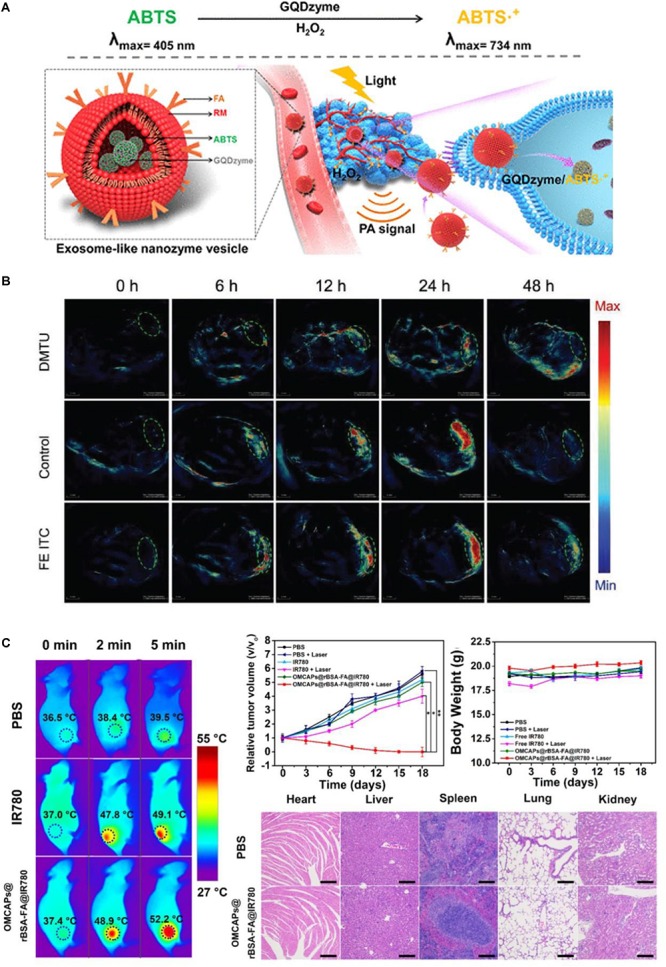
Nanozymes for *in vivo* imaging of disease progression. **(A)** Schematic illustration of exosome-like nanozyme vesicles for the H_2_O_2_-responsive catalytic photoacoustic imaging of tumors. Reproduced with permission from ref (Ding et al., 2019), © 2019, American Chemical Society. **(B)** Representative nanozyme-based tumor photoacoustic imaging images. Reproduced with permission from ref (Liu F. et al., 2019), © 2019, John Wiley and Sons. **(C)** Carbon-gold hybrid nanozymes for real-time imaging, photothermal/photodynamic and nanozyme oxidative therapy of tumors. Reproduced with permission from ref (Zhang et al., 2019).

In addition to enzyme-mimicking activity, nanozymes also exhibit fluorescence, electricity, paramagnetic properties and other unique physicochemical properties. By employing the unique physicochemical properties, nanozymes also have been broadly developed for *in vivo* monitoring and imaging of disease. For example, we utilized the unique *r2* relaxivity of iron nanozymes and achieved tumor *in vivo* magnetic resonance imaging (MRI) after we achieved the *in vitro* tumor tissue imaging by using the peroxidase-like activity of iron-based nanozymes with a single-dose of ^125^I-M-HFn NPs containing 45 μg HFn, 500 μCi ^125^I, and 11.2 μg Fe by intravenously injection (Zhao et al., 2016). We also designed a quantum-dot-based nanozyme vesicle for *in vivo* H_2_O_2_-responsive catalytic photoacoustic imaging of nasopharyngeal carcinoma (Ding et al., 2019). In this work, graphene quantum dots showed intense peroxidase activity and effectively catalyzed the peroxidase substrate 2,2′-azino-bis (3-ethylbenzthiazoline-6-sulfonic acid) diammonium salt (ABTS) into its oxidized form. The oxidized ABTS then exhibited strong near-infrared (NIR) absorbance, rendering it to be an ideal contrast agent for photoacoustic imaging. In the study, GQDzyme was at a dose of 100 μg/mL, 100 μL by an intravenously injection (Figure 3A). Jiang X. et al. (2018) achieved tumor phototherapy and simultaneous photoacoustic/thermal imaging and computed tomography by using a developed iridium oxide catalase nanozyme with extraordinary photothermal conversion efficiency and X-ray absorption coefficient showing a typical example of fully exploiting the multifunctional properties of nanozymes for tumor imaging and treatment. The BSA-IrO_2_ NPs used in the study was 1.5 mM, 200 μL via an intravenously injection (Jiang X. et al., 2018).

Nanozyme probes have also been broadly developed for disease therapeutic monitoring. For example, in 2019, Chen’s group prepared a tumor-microenvironment-activated nanozyme-mediated theranostic nanoreactor for imaging-guided combined tumor therapy (Liu F. et al., 2019). In their work, the constructed activatable nanoreactors achieved non-invasive imaging of tumor progression by using nanozyme-mediated photoacoustic imaging signal and photothermal therapy (PTT) function and the AMP NPs were at a dose of 10 mg/kg, 200 μL (Figure 3B). Cui’s group also prepared a mesoporous carbon-gold hybrid nanozyme probe for real-time imaging, photothermal/photodynamic and nanozyme oxidative therapy of tumors (Zhang et al., 2019). The results demonstrated that the synthesized nanozyme probes revealed excellent tumor targeting efficacy, long tumor retention, and favorably diagnostic and therapeutic effect for tumor (Figure 3C).

Besides cancer imaging diagnosis, nanozymes also have been broadly exploited for many other disease imaging such as infections, inflammation and some neurological diseases. For example, Rotello et al., reported a gold NPs-based charge-switchable nanozyme for bioorthogonal imaging of biofilm-associated infections (Tonga et al., 2015). In this work, the developed gold nanozymes could penetrate and accumulate inside the acidic microenvironment of biofilms and achieved imaging detection of the biofilm-associated infections arising from different and/or mixed bacteria species. Zhao et al. fabricated MnO_2_ nanozymes as the intracellular catalytic DNA circuit generators for versatile imaging of DNA base-excision repair in living cells (Chen et al., 2017). MnO_2_ nanosheet was used not only as a DNA nano-carrier but also a DNAzyme cofactor supplier. In this study, DNAzyme strands are blocked via the hybridization with the damaged bases-containing excision probes, which could be recognized by the corresponding base-excision repair enzymes in living cell. The detection signal could be 40-fold amplified by integrating several sets of probes with a dose of 20 μg mL^–1^ MnO_2_ nanozymes. Likewise, Yang et al. (2018) reported a nanozyme tag enabled chemiluminescence imaging probe for simultaneous multiplex imaging of cytokines. The prepared chemiluminescence nanozyme probe provides a novel and universal nanozyme-labeled multiplex immunoassay strategy for high-throughput detection of relevant biomarkers and further disease diagnosis. Thus, nanozymes open novel avenues for monitoring the initiation and progress of diseases by combining the unique physicochemical properties and enzyme-like catalytic activities of nanozymes.

## Summary and Outlook

The emergence of nanozymes uncovers the biological effects of inorganic nanomaterials. Nanozymes thus can be used as an alternative of natural enzymes because of their capability to address the limitations of natural enzymes such as low stability, high cost, and difficult storage. Over the past decade, nanozyme-based probes have been widely developed for disease imaging and diagnosis from *in vitro* to *in vivo*. The typical nanozymes for disease imaging diagnosis are summarized in Supplementary Table S1.

Despite the remarkable advantages of nanozymes, there still remains plenty of limitations while put nanozymes into practical clinical application, such as poor dispersibility, easy sedimentation after surface modification, limited catalytic types, poor substrate selectivity, and potential nanotoxicity. To further drive the rapid development of nanozyme-based imaging agents for disease diagnosis, substantial breakthroughs are expected by overcoming the following challenges: (1) Rational design and surface modification are still remain critical challenges to improve their substrate selectivity and dispersibility of nanozymes. Thus, both experimental and computational studies should be combined together to aid in the process of nanozyme design and surface modification. (2) A detailed understanding of the relationship between the catalytic properties and the *in vivo* biological behaviors of nanozymes is necessary. It is because the size, morphology and surface property of nanozymes have a direct impact on their catalytic activity and thus determine the *in vivo* biological behaviors of nanozymes. (3) A systematic evaluation of the biological fates and the biocompatibility of nanozyme systems (including the cytotoxicity, *in vivo* uptake and behavior, biodistribution, immunogenicity, blood compatibility, and tissue compatibility) should be performed when administrated into living organisms, since the biocompatibility and biodegradability are the common concerns when move these systems into clinical practice. (4) Developing standards and reference materials. Although nanozymes have shown a broad range of applications from *in vitro* biological detection to *in vivo* imaging diagnosis, there are rare basic concepts and the corresponding standards on nanozyme research. Therefore, nanozymes performance should be fully normalization according to their size, shape, modification to compare with each other when used for disease imaging diagnosis.

## Author Contributions

PW, TW, ML, and XY researched the literature and wrote the review. All authors revised and polished the review.

## Conflict of Interest

The authors declare that the research was conducted in the absence of any commercial or financial relationships that could be construed as a potential conflict of interest.
