# Supplementary material for: Nanozymes: A New Disease Imaging Strategy
Source: Front Bioeng Biotechnol. 2020 Feb 6;8:15. doi: 10.3389/fbioe.2020.00015 (PMC7015899; doi:10.3389/fbioe.2020.00015)
Supplement: Supplementary file 1 [file Table_1.DOCX]

**Supplementary table1: The typical nanozymes for disease imaging**

| **Nanomaterials** | | **Physical characteristics** | **Enzyme-**  **mimicking activity** | **Bio-**  **markers** | **Disease** | **Ref** |
| --- | --- | --- | --- | --- | --- | --- |
| Metal  oxide | Fe_3_O_4_ | Magnetism | POD | CSPG | Melanoma  CTCs | [1] |
|  | Fe_3_O_4_ | MRI | POD | ---- | Tumor theranostic | [2] |
|  | M-HFn | Targeting | POD | TfR1 | Tumor tissue， high-risk plaque tissues | [3, 4] |
|  | M-HFn | MRI | POD | ---- | Visualization of breast cancer cells | [5] |
|  | Fe_3_O_4_@Pt | Magnetism | POD | HER2 | Point-of-care bioassay | [6] |
|  | Fe_3_O_4_ /rGO | Magnetism | POD | ACh | Neuropsychiatric disorders | [7] |
|  | Co_3_O_4_ | ---- | POD | VEGFR | Tumor tissue | [8] |
|  | Fe-PDAP NFs | MRI | CAT | ---- | Multimodal tumor theranostic | [9] |
|  | CePO_4_：Tb, Gd nanospheres | MRI and fluorescent imaging | POD | ---- | Multimodal  imaging | [10] |
|  | MnO | MRI | SOD | ---- | Tumor theranostics | [11] |
|  | MnO_2_ | DNAzyme cofactor supplier | ---- | ---- | Living cell BER pathway | [12] |
|  | MnO_2_ nanosheet | ---- | CAT | ---- | UCL/PDT/RT imaging | [13] |
|  | CuO | CL | POD | CEA | Tumor diagnosis | [14] |
|  | Gd(OH)_3_ and Gd_2_O_3_ nanorods | MRI | POD | L‐cysteine | Cardiovascular and neurotoxic disease | [15] |
|  | Prussian blue | MRI | CAT | H_2_O_2_ | Ultrasound imaging | [16] |
| Noble metal | Ag | SERS | POD | CRP | Inflammatory | [17] |
|  | Ag | Dark-field imaging | POD | HER2 | Quantitative analysis of cancerous tissue | [18] |
|  | Au | Localized SPR | GOx | ATP | Real-time imaging of targets | [19] |
|  | Au | Two-photon photo-luminescence | POD | Integrin GPIIb/IIIa | Quantification of membrane proteins on the cell surface | [20] |
|  | PtCo | Magnetism | OXD | ---- | Cancer cell imaging | [21] |
|  | Au nanoclusters | Photostimulated enzyme mimetics | OXD | Trypsin | Pancreatitis | [22] |
|  | Au/Ag | ---- | POD | ACh | Parkinson’s and Alzheimer’s disease | [23] |
|  | Pt@mSiO_2_ | ---- | POD | BRCA1/2 | Breast cancer | [24] |
|  | PtNPs | ---- | CAT | BNP，CEA.et.al | Point-of-care diagnostics | [25] |
| Composite  Nano-  materials | Au-Fe_3_O_4_ | Fluorescence and MRI | POD | ---- | Dual modal imaging cancer cells | [26] |
|  | GO-Fe_3_O_4_ | Magnetism | POD | Glucose | Diabetic | [27] |
|  | FA-PtNPs/GO | ---- | POD | FAR | MCF-7 cancer cell imaging | [28] |
|  | CoxFe_3-x_O_4_ | Magnetism | POD  | DA | Schizophrenia | [29] |
|  | Fe_3_O_4_@MIL-100(Fe) | Magnetism | POD  | Cholesterol | Coronary heart, myocardial infarction and stroke | [30] |
|  | Ag@Au-Fe_3_O_4_ | Magnetism | POD  | Human IgG | Protein biomarker  detection | [31] |
|  | ZnFe_2_O_4_@MWNTs | Magnetism | POD | CEA | Tumor diagnosis | [32] |
|  | Prussian blue/manganese dioxide | MRI, PA imaging and PT imaging | CAT |  | Oxygen regulation of the xenografted breast cancer | [33] |
|  | V_2_O_5_‐PDA‐Au NPs | ---- | POD, GOx | Glucose | Diabetes | [34] |
|  | NaYF4:Yb,Er |  | POD | Uric acid | Hyperuricemia, renal impairment and liver disease | [35] |
|  | Fe-Co | ---- | POD | Glucose | Diabetes | [36] |

**Abbreviations**

POD：peroxidase

OXD：oxidase

CAT：catalase

SOD：superoxide dismutase

GOx：glucose oxidase

CSPG：chondroitin sulfate proteoglycan

CTCs：circulating tumor cells

TfR1：transferritin receptor 1

MRI：magnetic resonance imaging

HER2: human epithelial growth factor receptor-2

ACh：acetylcholine

BRCA1/2: breast cancer gene1/2

VEGFR：vascular endothelial growth factor receptor 2

BER pathway：base-excision repair pathway

UCL：upconversion luminescent

SPR：surface plasmon resonance

RT：radiation therapy

CL：chemiluminescent

CEA：carcinoembryonic antigen

SERS: surface enhancement of raman scattering

CRP: human C-reactive protein

SPR：surface plasmon resonance

BNP: B-type natriuretic peptide

FA：folic acid

FAR：folic acid receptor

DA：dopamine

PA：photoacoustic

PT：photothermal

**References**

1. Li, J., et al., *Simple and rapid colorimetric detection of melanoma circulating tumor cells using bifunctional magnetic nanoparticles.* Analyst, 2017. **142**(24): p. 4788-4793.

2. Zhang, D., et al., *Anti-bacterial and in vivo tumor treatment by reactive oxygen species generated by magnetic nanoparticles.* Journal of Materials Chemistry B, 2013. **1**(38): p. 5100-5107.

3. Fan, K., et al., *Magnetoferritin nanoparticles for targeting and visualizing tumour tissues.* Nature Nanotechnology, 2012. **7**(7): p. 459-64.

4. Wang, T., et al., *Bioengineered magnetoferritin nanozymes for pathological identification of high-risk and ruptured atherosclerotic plaques in humans.* Nano Research, 2019. **12**(4): p. 863-868.

5. Cai, Y., et al., *Enhanced magnetic resonance imaging and staining of cancer cells using ferrimagnetic H-ferritin nanoparticles with increasing core size.* International journal of nanomedicine, 2015. **10**: p. 2619-2634.

6. Kim, M.S., et al., *Pt-decorated magnetic nanozymes for facile and sensitive point-of-care bioassay.* ACS Applied Materials & Interfaces, 2017. **9**(40): p. 35133-35140.

7. Qian, J., et al., *Facile preparation of Fe_3_O_4_ nanospheres/reduced graphene oxide nanocomposites with high peroxidase-like activity for sensitive and selective colorimetric detection of acetylcholine.* Sensors and Actuators B: Chemical, 2014. **201**: p. 160-166.

8. Dong, J., et al., *Co_3_O_4_ nanoparticles with multi-enzyme activities and their application in immunohistochemical assay.* ACS Applied Materials & Interfaces, 2014. **6**(3): p. 1959-1970.

9. Bai, J., et al., *A facile ion-doping strategy to regulate tumor microenvironments for enhanced multimodal tumor theranostics.* Journal of the American Chemical Society, 2018. **140**(1): p. 106-109.

10. Wang, W., X. Jiang, and K. Chen, *CePO_4_:Tb,Gd hollow nanospheres as peroxidase mimic and magnetic–fluorescent imaging agent.* Chemical Communications, 2012. **48**(54): p. 6839-6841.

11. Ragg, R., et al., *Intrinsic superoxide dismutase activity of MnO nanoparticles enhances the magnetic resonance imaging contrast.* Journal of Materials Chemistry B, 2016. **4**(46): p. 7423-7428.

12. Feng, C., et al., *Fabricating MnO_2_ nanozymes as intracellular catalytic DNA circuit generators for versatile imaging of base-excision repair in living cells.* Advanced Functional Materials, 2017. **27**(45): p. 1702748.

13. Fan, W., et al., *Intelligent MnO_2_ nanosheets anchored with upconversion nanoprobes for concurrent pH-/H_2_O_2_-responsive UCL imaging and oxygen-elevated synergetic therapy.* Advanced Materials, 2015. **27**(28): p. 4155-4161.

14. Li, J., et al., *Efficient label-free chemiluminescent immunosensor based on dual functional cupric oxide nanorods as peroxidase mimics.* Biosens Bioelectron, 2018. **100**: p. 304-311.

15. Singh, M., et al., *Competitive inhibition of the enzyme-mimic activity of Gd-based nanorods toward highly specific colorimetric sensing of l-Cysteine.* Langmuir, 2017. **33**(38): p. 10006-10015.

16. Yang, F., et al., *A hydrogen peroxide-responsive O_2_ nanogenerator for ultrasound and magnetic-resonance dual modality imaging.* Advanced Materials, 2012. **24**(38): p. 5205-5211.

17. Sloan-Dennison, S., et al., *A novel nanozyme assay utilising the catalytic activity of silver nanoparticles and SERRS.* Analyst, 2017. **142**(13): p. 2484-2490.

18. Lin, F., et al., *Enzyme catalysis enhanced dark-field imaging as a novel immunohistochemical method.* Nanoscale, 2016. **8**(16): p. 8553.

19. Liu, Q., et al., *Nanoplasmonic detection of adenosine triphosphate by aptamer regulated self-catalytic growth of single gold nanoparticles.* Chemical Communications, 2012. **48**(77): p. 9574-9576.

20. Gao, L., et al., *Peptide-conjugated gold nanoprobe: intrinsic nanozyme-linked immunsorbant assay of integrin expression level on cell membrane.* ACS Nano, 2015. **9**(11): p. 10979-90.

21. Cai, S., et al., *PtCo bimetallic nanoparticles with high oxidase-like catalytic activity and their applications for magnetic-enhanced colorimetric biosensing.* Journal of Materials Chemistry B, 2016. **4**(10): p. 1869-1877.

22. Wang, G.L., et al., *Intrinsic enzyme mimicking activity of gold nanoclusters upon visible light triggering and its application for colorimetric trypsin detection.* Biosensors and Bioelectrontics, **64**: p. 523-529.

23. Wang, C.-I., W.-T. Chen, and H.-T.J.A.c. Chang, *Enzyme mimics of Au/Ag nanoparticles for fluorescent detection of acetylcholine.* Analytical Chemistry 2012. **84**(22): p. 9706-9712.

24. Paul, A. and S.J.F.B. Paul, *The breast cancer susceptibility genes (BRCA) in breast and ovarian cancers.* Front Biosci, 2014. **19**(4): p. 605-618.

25. Li, Y., et al., *A microfluidic platform with digital readout and ultra-low detection limit for quantitative point-of-care diagnostics.* Lab on a Chip,2015. **15**(16): p. 3300-3306.

26. Liu, J., et al., *A multifunctional nanoprobe based on Au–Fe_3_O_4_ nanoparticles for multimodal and ultrasensitive detection of cancer cells.* Chemical Communications, 2013. **49**(43): p. 4938-4940.

27. Dong, Y.L., et al., *Graphene oxide-Fe_3_O_4_ magnetic nanocomposites with peroxidase-like activity for colorimetric detection of glucose.* Nanoscale, 2012. **4**(13): p. 3969-76.

28. Zhang, L.-N., et al., *In situ growth of porous platinum nanoparticles on graphene oxide for colorimetric detection of cancer cells.* Analytical Chemistry, 2014. **86**(5): p. 2711-2718.

29. Niu, X., et al., *Visual and quantitative determination of dopamine based on CoxFe_3−x_O_4_ magnetic nanoparticles as peroxidase mimetics.* Journal of Alloys and Compounds, 2014. **587**: p. 74-81.

30. Wu, Y., et al., *Metal-organic framework coated Fe_3_O_4_ magnetic nanoparticles with peroxidase-like activity for colorimetric sensing of cholesterol.* Sensors and Actuators B: Chemical, 2017. **249**: p. 195-202.

31. Zhang, H., et al., *A novel electrochemical immunosensor based on nonenzymatic Ag@Au-Fe_3_O_4_ nanoelectrocatalyst for protein biomarker detection.* Biosensors and Bioelectronics, 2016. **85**: p. 343-350.

32. Liu, W., et al., *Paper-based colorimetric immunosensor for visual detection of carcinoembryonic antigen based on the high peroxidase-like catalytic performance of ZnFe_2_O_4_–multiwalled carbon nanotubes.* Analyst, 2014. **139**(1): p. 251-258.

33. Peng, J., et al., *“One-for-All”-type, biodegradable prussian blue/manganese dioxide hybrid nanocrystal for trimodal imaging-guided photothermal therapy and oxygen regulation of breast cancer.* ACS Applied Materials & Interfaces, 2017. **9**(16): p. 13875-13886.

34. Qu, K., et al., *Nanocomposite incorporating V_2_O_5_ nanowires and gold nanoparticles for mimicking an enzyme cascade reaction and its application in the detection of biomolecules.* Chemistry-A European Journal, 2014. **20**(24).

35. Yu-Rong, T., et al., *Application of NaYF_4_: Yb, Er nanoparticles as peroxidase mimetics in uric acid detection.* Chinese Journal of Analytical Chemistry, 2013. **41**(3): p. 330-336.

36. Chen, Y., et al., *Fe–Co bimetallic alloy nanoparticles as a highly active peroxidase mimetic and its application in biosensing.* Chemical Communications, 2013. **49**(44): p. 5013-5015.
